# Study of Health in Primary Care of the Amazonas Population: Protocol for an Observational Study on Diabetes Management in Brazil

**DOI:** 10.2196/37572

**Published:** 2022-09-15

**Authors:** Elisa Brosina de Leon, Hércules Lázaro Morais Campos, Fabiana Almeida Brito, Fabio Araujo Almeida

**Affiliations:** 1 Programa de Pós Graduação em Ciências do Movimento Humano Faculdade de Educação Física e Fisioterapia Universidade Federal do Amazonas Manaus Brazil; 2 Instituto de Saúde e Biotecnologia Universidade Federal do Amazonas Coari Brazil; 3 Department of Health Promotion College of Public Health University of Nebraska Medical Center Omaha, NE United States

**Keywords:** health management, diabetes, patient activation, health care, T2DM, Amazon, patient profile, behavioral change, health policy, epidemiological profile, epidemiology, management

## Abstract

**Background:**

Changes in the profiles of patients have significant impacts on the health care system. Diabetes mellitus type 2 (T2DM) prevention and management should be studied in different contexts.

**Objective:**

The Study of Health in Primary Care for the Amazonas Population (SAPPA) primarily aims to describe T2DM prevention and management actions offered by primary health care settings in Brazil and whether the care delivered is consistent with the chronic care model (CCM). Second, the study aims to examine the impact of T2DM management actions on health and lifestyle, and third, to understand how sociodemographic characteristics, health, and subjective outcomes impact diabetes management.

**Methods:**

As part of this observational study, managers and health professionals complete a questionnaire containing information about T2DM prevention and management actions and CCM dimensions. During in-home visits, patients are asked about their health, lifestyle, sociodemographics, diabetes care, and subjective variables.

**Results:**

A total of 34 managers, 1560 professional health workers, and 955 patients will be recruited. The data collection will be completed in October 2022.

**Conclusions:**

The SAPPA is an observational study that intends to understand the T2DM management process in primary health care, including planning, execution, reach, and impact on patient motivation and adherence.

**International Registered Report Identifier (IRRID):**

DERR1-10.2196/37572

## Introduction

The increase in life expectancy of the global population entails a growing demand for health care, due to increases in noncommunicable chronic diseases added to communicable diseases [1]. The transformation in the profile of patients means a new approach to health care [2]. However, there is a mismatch between the increase in chronic conditions and the management process, which still privileges acute conditions or the worsening of chronic conditions [3]. Any changes face barriers of verticality, immobilization, and fragmentation of the health system [4].

It has also been observed that health systems, in addition to having an essential role in the prevention of chronic diseases and in long-term health management strategies, must be developed alongside other sectors and community actors [5]. The care model adopted is crucial for the success of improving the living conditions of an individual or a community. In this case, the chronic care model (CCM) [6] is highlighted.

The CCM is based on the interaction between active and informed patients and health care teams that are proactive and prepared to meet the demands of the population under their responsibility. This model recommends that the highest care organization depends on key elements linked to the health care system, including health system/organizational support, clinical information systems, delivery system design, decision support, self-management support, and community resources [7].

Additionally, health care must consider institutional arrangements to meet populational, family, and individual demands, responding to specific contexts and respecting people’s unique needs [6]. The macrolevel of health systems must also consider the microlevel of each patient, to optimize trajectories of intrinsic capacity [8]. There is evidence that focusing on the intrinsic capacity is more effective than prioritizing the treatment of specific chronic diseases [9,10]. This does not mean rejecting the treatment, but rather emphasizes that the preservation and recovery of people’s physical and mental capacities must be objectives and starting points for health interventions. This proposition makes sense when we consider that people with strengthened physical and mental capacities will be better conditioned to respond to losses caused by the aging process and diseases. They may also respond better to rehabilitation or recovery processes in cases of injuries or acute illnesses [10].

Understanding that different contexts can influence the care provided and received by the population leads to the need to search for evidence that supports the best planning of strategies. Therefore, the Study of Health in Primary Care of the Amazonas Population (SAPPA) is being conducted in the Amazonas Population. Three main aims motivated this research. The first aim is to describe management actions offered in primary health care settings in the State of Amazonas, Brazil, and whether the care delivered is consistent with the CCM. The second aim is to examine how this assistance addressed to patients with type 2 diabetes mellitus (T2DM) impacts health and lifestyle. The third aim is to better understand how sociodemographic characteristics, health, and diabetes and subjective variables could impact diabetes management.

## Methods

### Design Overview

**Figure 1:**
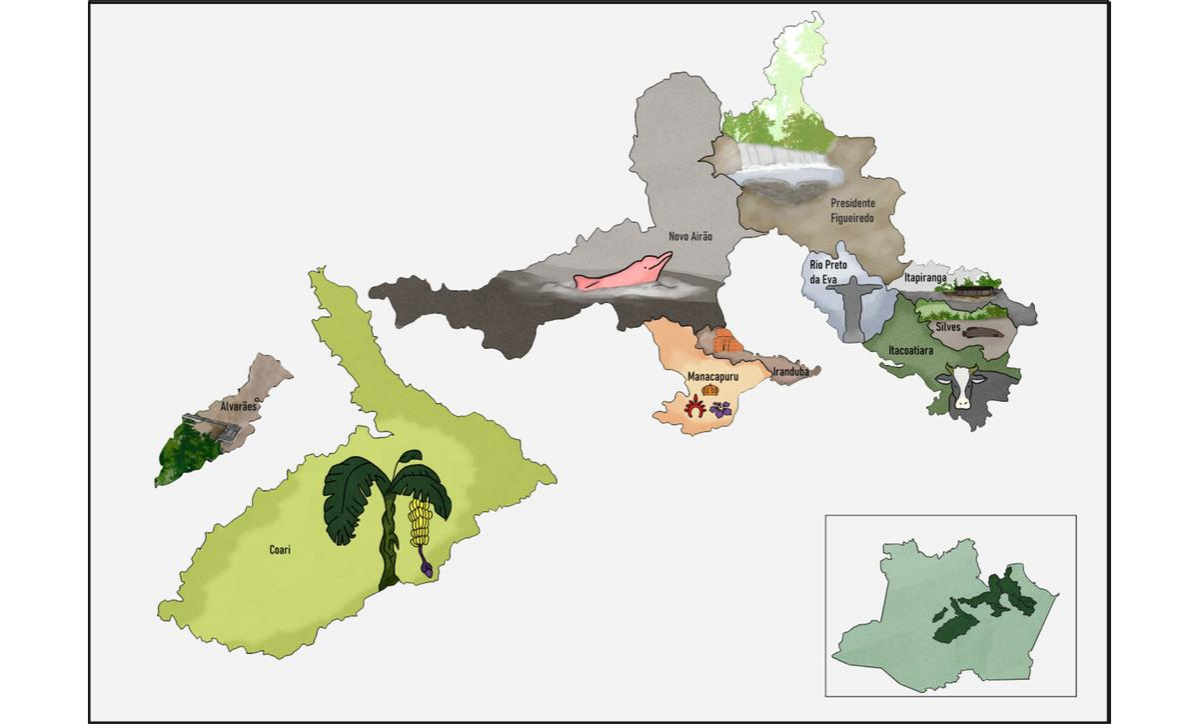
Amazonas cities.

The SAPPA is an observational study carried out on the population of Amazonas, Brazil. Data collection is being conducted in 2 Amazonas regions. The first region includes cities in the metropolitan region of Manaus. In 2010, this region had a degree of urbanization of 94% and approximately 60% of the state population resided in this area [11]. The metropolitan region of Manaus includes Iranduba (38.1 km by car from the capital), Itacoatiara (270 km by car from the capital), Manacapuru (98.8 km by car from the capital), Novo Ayrão (194.8 km by car from the capital), Presidente Figueiredo (125.5 km by car from the capital), Rio Preto da Eva (80.2 km by car from the capital), Silves (267 km by car from the capital), and Itapiranga (339.1 km by car from the capital). The second region of SAPPA includes cities located at Medium Solimões: Coari (363 km by boat from the capital) and Alvarães (532 km by boat from the capital; Figure 1).

### Setting

Briefly, the Brazilian health system is elaborated from a set of institutional environments between the 3 spheres of management (union, states, and cities). This approach aims to promote innovations in management processes and instruments, proposing to achieve greater efficiency and quality in the responses of the unified health system [12].

In this process, the Health Ministry (federal government) is responsible for proposing policies; participating in the financing, technical cooperation, evaluation, regulation, control, and inspection of health care; and medication supply [13].

For T2DM, preventive and health promotion measures are integrated, including support for the prevention of complications, diagnosis of cases, treatment and follow-up, emergency care, and case referral. However, the responsibility for coordinating and planning actions based on the recommendations for the prevention and management of T2DM is held in primary health care settings [14].

**Figure 2:**
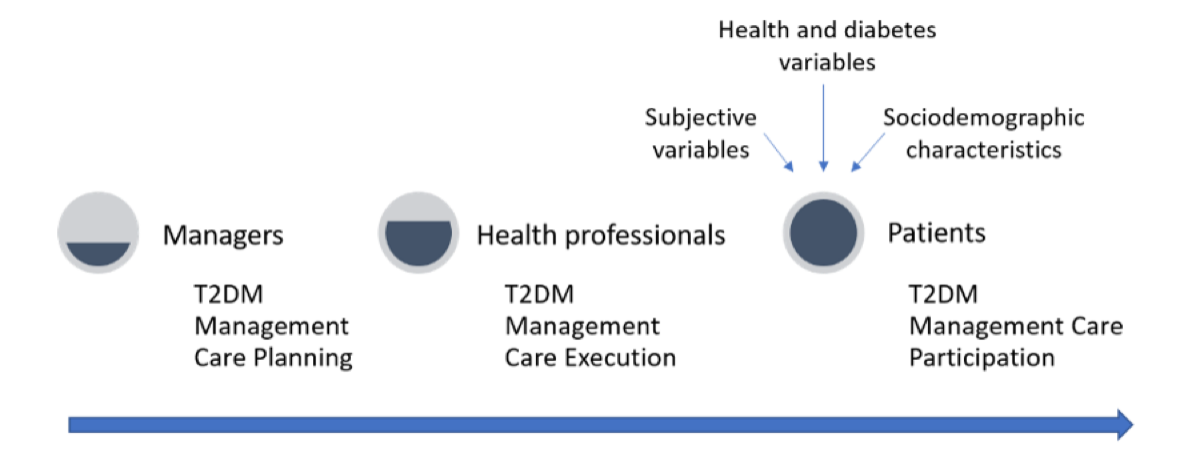
Diabetes management process in primary health care settings. T2DM: Diabetes mellitus type 2.

At the municipal level, the primary health care manager and health professionals are required to define the actions and services that should be developed in their settings and build the care flows that must be guaranteed to the patients in order to attend to patients’ health needs [14]. In this way, primary health care settings play a central role in the T2DM management process (Figure 2).

### Participant Eligibility and Recruitment

The SAPPA is being conducted in primary health care units in each city, including urban and rural areas, according to the National Register of Health Establishments. Selection takes place randomly by drawing lots, and all units have the same chance of being drawn. Recruitment of participants started in August 2020, and the data collection is intended to be completed in October 2022.

**Figure 3:**
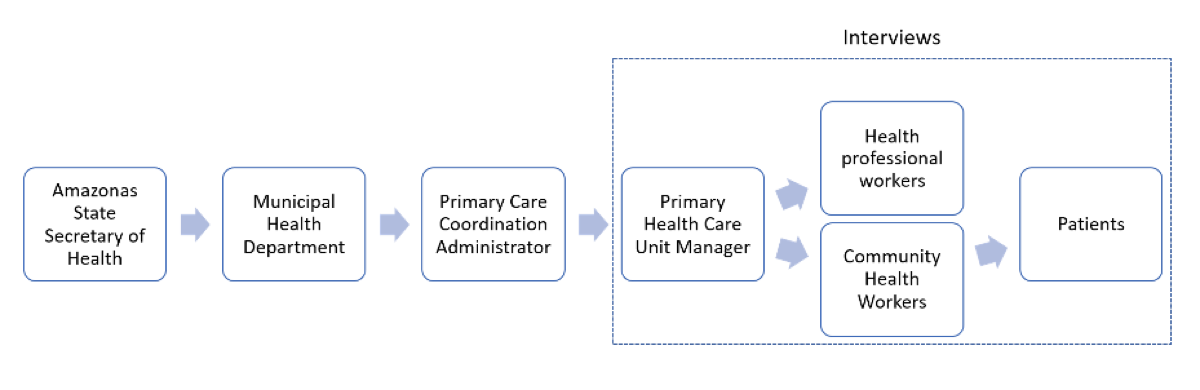
Consent and recruitment process.

Figure 3 describes the process for engaging cities and administrators in the study and recruitment. Briefly, initially, the Amazonas State Secretary of Health was contacted for approval. Following this approval, each municipal health department was contacted to engage administrators and managers. After agreement from the municipal health department to participate in the study, the primary health care coordination administrators for each municipality were contacted. These coordinators facilitate contact with managers from each primary health care unit. An individual member from the SAPPA contacts the primary health care unit managers by phone to schedule the site visit for data collection. During the site visit, the project proposal and the field team are presented to the community health workers to establish the partnership. The community health workers provide patients’ names and addresses and accompany the field team during home visits.

Inclusion criteria for managers: All managers from primary health care units are invited to participate and are interviewed in private in their offices.

Inclusion criteria for professional health workers: professional health workers who have been involved in patient assistance for at least 3 months, including nurses, health technicians, physicians, community health workers, physical educational professionals, physiotherapists, dieticians, and dentists are invited to participate in the study. Inclusion criteria for patients: patients with a T2DM diagnosis and in primary health care for at least 6 months.

Exclusion criteria for professional health workers: professional health workers who are on vacation or sick leave. Exclusion criteria for patients: patients who present a communication disorder that makes it unfeasible to collect data.

### Sample Size Calculation

Considering the total number of 142 health units distributed in urban and rural areas in 10 cities, and setting the margin of error at 15% and the confidence level at 95%, a sample of 34 units was obtained. Proportional stratified sampling was used for the estimated number of interviews per city. The distribution of the sample is detailed in Table 1. The choice of individuals and urban and rural units for collection was made using the simple random sampling method.

The SAPPA sampling design was based on the Continuous National Household Sample Survey, used to estimate the total number of the study population, thus satisfying the sampling proportionalities within each stratum. The target population was formed by patients with DM2 registered in primary health care in each city. The estimated number of T2DM patients was obtained considering the prevalence of diabetes of 5.2% [15]. This sample size was calculated for each city and allows a confidence level of 99% and a margin of error of 10%.

**Table 1:** Sample distribution.

City and location		Total primary health care units	Selected primary health care units	Patient interview calculation
**Iranduba**				
	Rural	5	1	26
	Urban	15	3	78
**Itacoatiara**				
	Rural	9	2	146
	Urban	7	1	73
**Itapiranga**				
	Urban	5	1	20
**Manacapuru**				
	Rural	9	2	84
	Urban	12	3	126
**Novo Airão**				
	Rural	5	1	21
	Urban	5	1	21
**Presidente Figueiredo**				
	Rural	14	3	47
	Urban	9	2	31
**Rio Preto da Eva**				
	Rural	10	2	48
	Urban	5	1	24
**Silves**				
	Rural	8	1	20
	Urban	2	0	0
**Alvarães**				
	Rural	3	1	18
	Urban	4	2	16
**Coari**				
	Urban	15	7	156
Total		142	34	955

### Data Collection Procedures

The field team is composed of 30 interviewers and 2 supervisors. The supervisors are part of the coordination team, and the interviewers are undergraduate students from the Health College (Nursing and Physical Therapy College). The field team was carefully trained before the start of the study by repeatedly applying SAPPA forms. Three interviewers were trained to exclusively apply the forms to managers and health workers. The other interviewers were trained to apply the forms to patients. Videos were specially produced for the training of anthropometric and functional capacity measurements. The field team training takes approximately 3 hours. To ensure the same measurement standards and practices, after training the interviewers conduct a mock session under staff supervision. A series of checks are conducted regularly to identify outliers and discrepancies in measurements. Appropriate measures are taken whenever problems are detected. If necessary, field team members are retrained, and equipment is constantly checked and replaced.

Data collection in cities in the metropolitan region of Manaus is performed in a round site visit. The field team for each visit includes 1 supervisor, 3 manager/health worker interviewers, and at least 12 patient interviewers. On a previously scheduled date, the field team leaves Manaus for the target cities using the transport from the University and carrying all the data collection equipment.

Upon arriving at the primary health care units, the supervisor introduces themself to the manager and presents the project proposal and field team to the professional health workers. The manager/professional interviewer remains at the primary health care unit for the interviews, while the other interviewers and the supervisor accompany the community health workers on home visits. At the patient residence, the interviewers are introduced to the patients by the community health workers and invite the patients to join the research.

After the patient agrees to participate in the research, data collection begins. The patient forms and evaluations are applied in the home by the field team, and take an average of 1 hour to complete. For the Medium Solimões data collection, a local field team was formed, including 1 supervisor, 2 manager/professional interviewers, and 12 patient interviewers. After training, the same data collection procedure was adopted.

### Instruments

Table 2 describes the variables included in the SAPPA data collection.

**Table 2:** Instruments for data collection.

Participants		
**Managers**		
		Development and execution of actions for the prevention and management of T2DM^a^
**Professional health workers**		
		Participation in actions for the prevention and management of T2DM Assistance of the organization with respect to T2DM evaluation
**Patients**		
	Sociodemographic characteristics	Sex, age, marital status, skin color, educational level, employment status, monthly income, number of household members, and religion
	Health-related variables	Self-perceived health Memory and cognition Patient engagement Disease burden Medications and drug use (including antidiabetic drugs and insulin) Frequency of consumption of medicinal plants Pain
	Physical frailty	Physical functional capacity Frailty Sarcopenia T2DM diagnosis time
	Diabetes variables	Participation in actions focused on diabetes management Nutritional behavior Quality of care
	Behavioral variables	Physical activity level and sedentary behavior Tobacco and alcohol consumption Insomnia and daytime sleep
	Subjective health assessment	Intrinsic religiosity Life purpose Overall life satisfaction
	Anthropometric measurements	Height, weight, and BMI

^a^T2DM: type 2 diabetes mellitus.

#### Manager Instruments

Managers are invited to complete a form containing information about T2DM prevention and management actions already implemented in the primary health care units. These forms are applied during the on-site visits by a field researcher using an Android device.

To identify actions taken to manage T2DM in primary health care in the State of Amazonas, the managers are asked to complete a questionnaire describing the development of prevention and management of T2DM actions, scope of the actions, cost of implementation and professionals involved, effectiveness, and maintenance of the actions [16].

#### Professional Health Worker Instruments

Professional health workers are interviewed by a field researcher in the primary health care settings. They are invited to respond to questions about their participation in actions related to the prevention and management of T2DM [16]. They then complete the questionnaire for the Assessment of Chronic Illness Care (ACIC).

To assess and monitor health care systems, researchers from the McColl Institute for Health Care Innovation [17] proposed 2 instruments: the ACIC and the Patient Assessment of Chronic Illness Care. The ACIC provides guidance for professional health workers in order to assess their perception of the care provided by their setting for T2DM chronic conditions.

The ACIC is composed of 6 dimensions associated with the realization of the CCM, and a seventh, which assesses the integration of the dimensions. The perceptions obtained are analyzed using a scoring scale, from 0 to 11, where 0 represents the lowest score, that is, a place with very limited resources and structures and 11, the highest score, indicating a place with good resources and an optimal structure for the care of chronic conditions. The 7 dimensions of the ACIC instrument are made up of the organization of health care, interaction with the community, supported self-care, decision support, design of the delivery system service, clinical information system, and integration of CCM components [18].

#### Patient Instruments

The patient is interviewed at home, through a form completed by a field researcher using an Android device. The following variables are collected:

Sociodemographic variables include sex, age, race/ethnicity, marital status, educational level, employment status, monthly income, number of household members, and religion.

Health-related variables included cognitive status, self-perceived health, disease burden, medications, consumption of medicinal plants, patient engagement, and pain.

- Cognitive assessment is addressed through the 10-point Cognitive Screener [19] and Figure Recognition Test [20], a brief and easy-to-use screening strategy with higher accuracy developed for the Brazilian population [19], presenting sensitivity of 60.5 and specificity of 94.3 [21].
- Self-perceived health is obtained through questions such as, “In general, how do you assess your health at present?” “How do you rate your health compared to other people your age?” “How do you rate your memory compared to other people your age?” “How do you rate your health today compared to 1 year ago?” and “How do you rate your activity today, compared to a year ago?” Possible responses were much worse, worse, equal, better, much better, and no answer [22].
- Disease burden is measured by the Functional Comorbidity Index which consists of a list of 18 comorbidities. The score is obtained by the sum of all comorbidities present and ranges from 0 to 18 [23].
- The number of medications and medication adherence is collected through the registration of all medications prescribed in the last medical appointment. In addition, participants are asked how many days of medication doses were missed in the previous 7 days [24].
- The consumption of medicinal plants is measured by type and frequency.
- Patient engagement is a construct that includes self-efficacy, behavior, and knowledge, and has been shown to predict a variety of health behaviors [25]. The Patient Activation Measure -13 (PAM-13) was applied, which includes measurements of the use of self-management services, the performance of self-management behaviors, and medication adherence [25]. The PAM-13 is a 13-item measure that assesses self-efficacy, behavior, and knowledge [25]. Item scores range from 0 to 4, with 0=not applicable, 1=strongly disagree, 2=disagree, 3=agree, and 4=strongly agree. Mean PAM-13 scores are then transformed into a score ranging from 0 to 100, where higher scores represent higher activation. PAM-13 scores are assigned to 1 of the 4 stages of activation, based on the Insignia Health guidelines. PAM level 1 refers to very low activation (0-47 points) meaning disengaged and overwhelmed; level 2 (47,1-55,1), becoming aware but still struggling; level 3 (55.2-67.0), taking action; and level 4, high activation (67-100), meaning maintaining behaviors and pushing further [26]. This measurement was validated for the Brazilian population [27].
- The presence of pain and intensity was measured using the visual analog scale and Faces Pain Scale, which allows the quantification of pain intensity using numbers on a scale from 0 to 10, where 0 indicates the minimum pain and 10 indicates the maximum pain possible [28]. The Face Scale indicates the intensity of pain according to the mimicry represented on each drawn face, with the expression of happiness corresponding to the classification no pain and the expression of maximum sadness corresponding to the classification maximum pain [29].

Diabetes variables: T2DM diagnosis time, use of antidiabetic drugs or insulin, active participation in activities focused on diabetes prevention and management offered by primary health care and partners [16], and quality of care.

- Quality of care is measured by the Patient Assessment of Chronic Illness Care instrument, congruent with the CCM, whereby patients report their experiences of the care delivery system. The survey includes 20 items and is sufficiently brief to use in many settings [30]. When paired with the ACIC, these tools can provide complementary consumer and provider assessments of important aspects of care for patients with chronic illness [31]. The survey was validated for the Brazilian population [18]. Respondents answer each item with a response from 1=almost never to 5=almost always. For scoring: patient activation: average of items 1-3, delivery system: average of items 4-6, goal-setting: average of items 7-11, problem solving: average of items 12-15, and follow-up: average of items 16-20 [31].

Physical frailty: functional capacity, daily and instrumental activities of daily living, sarcopenia, and frailty.

- Short Physical Performance Battery Test is used to assess functional capacity, through the following domains: gait speed at a normal pace, static balance in an orthostatic position, and strength of the lower limbs through observation if the participant can get up and sit in a chair. Total scores are calculated by summing the 3 individual test items, with a potential range of 0-12 points. Higher scores indicate better lower body function [32], presenting a sensitivity of 79.7 and specificity of 73.8 [33].
- Brazilian version of the Older Americans Resources and Services Multidimensional Functional Assessment Questionnaire assesses the difficulty reported in performing 15 daily living and 7 instrumental activities of daily living. The total number of activities of daily living that the patient reports difficulty in performing is quantified, that is, the total number of activities compromised. The higher the score, the greater the impairment in functional capacity [34]. The tool presents sensitivity of 83 and specificity of 69 [35].
- The risk of sarcopenia is identified through the Strength, Ambulation, Rising from a Chair, Stair Climbing, and History of Falling questionnaire: muscle strength, the need for assistance with walking, ability to get up from a chair and climb stairs, and frequency of falls. The score given to each item is from 0 to 2 points, reaching the sum of 0 to 10 points. Patients who present a result greater than or equal to 4 in this questionnaire are classified as at risk of sarcopenia [36]. The circumference calf measurement is also included [37]. The tool presents sensitivity of 60 and specificity of 80.92 [38].
- Frailty will be addressed according to the Fried criteria [39]. These criteria include 5 components: (a) unintentional weight loss of ≥4.5 kg in the last year (obtained from the patient), (b) weakness (assessed using the hand-grip strength measurement; considering the interpretation of results based on sex and BMI), (c) exhaustion (evaluated based on 2 questions from the Center for Epidemiological Studies Depression scale [40]), (d) slow gait (walking time over a distance of 4 meters; interpretation of results takes into account sex and height), (e) low physical activity (weekly energy expenditure rate calculated based on International Physical Activity Questionnaire [IPAQ] [41]). An Instrutherm@ digital dynamometer is used for the hand-grip strength measurement.
- The Clinical-Functional Vulnerability Index–20 is used as a 20-item instrument for screening frailty developed by a Brazilian team that assess self-perception of health, activities of daily living, cognition, mood, mobility, communication, and multiple comorbidities, resulting in a classification based on the score achieved as follows: robust older adult when the score is 0 to 6, older adults at risk of frailty with a score between 7 and 14, and frail older adults when the score is greater than or equal to 15 [42]. The tool presents sensitivity of 90.5 and specificity of 71 [43].

Behavioral variables: nutritional behaviors, physical activity behaviors, tobacco and alcohol consumption, insomnia, and daytime sleep.

- Nutritional behavior (regular consumption of fruits and vegetables and fish).
- Physical activity behaviors: physical activity is measured using the short version of the IPAQ, validated for the Brazilian population [41]. Duration (minutes) and frequency (days) of physical activity in the previous 7 days is measured in the following domains: job-related, transportation, housework, house maintenance, caring for family, recreation, sport and leisure time, and time spent sitting. These activity categories are treated separately to obtain the specific activity patterns or multiplied by their estimated value in metabolic equivalent of tasks (METs) and summed to gain an overall estimate of physical activity in a week. The MET intensity values used to score IPAQ questions in this study are vigorous (8 METs), moderate (4 METs), and walking (3.3 METs) [44]. The IPAQ sitting question (2 questions) are indicators of the time spent in sedentary activity and not included as part of the summary score of total physical activity [41,45]. The IPAQ demonstrates sensitivity of 81 and specificity of 85 [46].
- Lifetime tobacco use is obtained through self-report with the question: “Do you have or did you have the habit of smoking?” (nonsmoker, ex-smoker, and current smoker) and “How long have you been a smoker?” [22].
- Alcohol consumption patterns are addressed by these questions: frequency of consumption (never drink alcoholic beverages; drink once a month; drink once a week; daily binge drinking) and amount ingested. High intake is defined as more than 5 units (bottle/can/dose/cup) on the same day [47].
- Insomnia and daytime sleep: questions were extracted from the Nottingham health profile and validated for use in Brazil [48]. The question on duration of daytime naps in the previous 12 months was taken from the Minnesota Leisure Activity Questionnaire [49].

Subjective assessment: intrinsic religiousness, life purpose, and overall life satisfaction.

- A brief measure of intrinsic religiousness, namely the Intrinsic Religiousness Inventory [50], a valid instrument to measure the impact of religion on physical and mental health in Brazilian samples [50,51], is applied. It consists of a structured questionnaire, which is organized on a Likert scale, with scores ranging from 1 to 5 that reflect gradations of intensity/frequency: 1=never, 2=rarely, 3=occasionally, 4=often, and 5=always. The initial version of the Intrinsic Religiousness Inventory was developed from the compilation of statements with themes related to the construct of intrinsic religiosity, based on a literature review and suggestions from specialists [50].
- The Brazilian version of the Life Purpose Scale is a 10-item self-report measure and was semantically and culturally validated for use with the Brazilian population [52].
- Overall life satisfaction questions: “Are you satisfied with: your life? Your health? Your memory to do and remember daily things? Your friendships and family relationships? The environment (climate, noise, pollution, attractions, and safety)?”

Anthropometric measurements: height, weight, and BMI.

- Height is measured to the nearest 1 mm using a tape measure with the participant in a full standing position (in the Frankfort horizontal plane). Patients are asked to wear light clothing and no shoes and the average of 2 measurements is considered.
- Weight is measured using a digital scale (model Digital Glass 7 FW, G-TECH) and the average of 2 measurements is considered.
- BMI is calculated from weight (kg) divided by the square of height (m).

### Data Collection Tools

The first step of the SAPPA data collection is the selection and preparation of the questionnaires included in each form: managers, professional health workers, and patients.

The forms for managers and health workers were created using a mobile-based data collection tool designed by KoBoToolbox (Kobo Inc). The field team carries an Android device during the interviews. The Android device (phone or tablet) is embedded with KoBoCollect and allows the forms to be constructed and reviewed entirely offline, as well as allowing data collection offline. The information system requires internet access and is performed by downloading the SAPPA questionnaire from the mobile-based data collection.

KoBoCollect is a data collection Android app based on an open data kit (ODK) that can be installed on any standard Android device (phone or tablet). The form captures the data and securely transmits it to the central server via Wi-Fi. Other alternative data transfer methods include mobile phone networks or a direct cable. One of the major advantages of the SAPPA information system is that all data are immediately available after data collection. Data storage and management are centralized at the Federal University of Amazonas using a cloud service.

The patient form was also built on ODK as previously described. To create a data collection flow during the home visit to the patient, the questions are grouped by categories: (1) sociodemographic variables, (2) cognitive evaluation, (3) religiousness, (4) health-related variables, (5) diabetes variables, (6) medications and medicinal plants, (7) pain, (8) behavior variables, (9) functional activities, (10) emotional aspects, (11) relationship, and (12) functional evaluation. The SAPPA questionnaire was built using a mobile-based data collection tool designed by KoBoToolbox.

### Ethics Approval

The SAPPA was approved by the Federal University of Amazonas ethics board (4.318.325 and 4.994.196), and the study was performed following approved guidelines and the Declaration of Helsinki. All participants are required to sign informed consent forms prior to interviews (managers, health workers, and patients), physical measurements (patients), and photo registry (patients).

Study findings will be disseminated through scientific conferences, peer-reviewed journal publications, and invited lectures. Results from this study will be reported according to Strengthening the Reporting of Observational Studies in Epidemiology (STROBE) guidelines and submitted for peer-reviewed publications.

### Statistical Analysis

Data will be analyzed using SPSS (version 20.0, IBM Corp). Unless otherwise specified, a significance level of *P*<.05 will be adopted in all statistical analyses. Sample and subsample characteristics will be presented using frequencies for categorical variables and mean and standard deviation for continuous variables. Multiple linear (continuous dependent variable) and logistic (binomial dependent variable) regression analyses will be used to study the association between the independent and dependent variables of interest. Other analyses may be applied and will be reported in future papers.

## Results

A total of 34 managers, 1560 professional health workers, and 955 patients will be recruited. The data collection is intended to be completed in October 2022.

## Discussion

We present the design of the ongoing SAPPA Study. The goal of the study is not only to build an understanding of the current T2DM management provided to the Amazonas population but also to provide scientific data to support policy changes that may affect and improve patient assistance. Identifying the current management process could allow us to propose a new approach for this unique population based on the SAPPA findings.

Cities in Amazonas are particular in several aspects when compared to other cities in Brazil. This is the case in the metropolitan region of Manaus, which has a peculiar spatiality composed of a large territorial extension with significant population gaps between its urban centers. Apart from Manaus, the other cities included have the characteristic of being responsible for supplying agricultural products and labor for the capital [53]. These cities, despite being close to the capital, have a much lower degree of development and great economic and social fragility, due to the scarcity of income-generating agents and the ease of migration. This situation is aggravated by moving away from the urban perimeter of these cities toward the various rural communities that are located on the riverbanks or roads and neighboring areas. In these places, access to goods and services becomes very difficult, expectations decrease, and social risks increase [54]. These inequalities reflect in the care conditions for the population, affecting the quality of care and life available for patients and assisted families [54].

Despite continuous investments, the findings of the study proposed by Silva et al [55] show a social scene that is ineffective for the health demands of the Amazonas population. The study demonstrates a predominance of asymmetry, verticality, competitiveness, and fragility in the assistance provided. Political agents involved in the process have limited understanding of the sociopolitical and institutional conditions in which they operate. They tend to attribute the management and operationalization problems of assistance networks to the configuration of Amazonian natural geographic spaces, but their financing, governance, and technical capacity are insufficient to overcome these problems [55].

The persistent absence of a care design capable of guaranteeing the referral of patients in the health region perpetuates an informal flow in which situations are resolved on a case-by-case basis, without a definition of priorities and a cost-effectiveness analysis to properly direct the resolution. The lack of discussions and agreements aimed at instituting care networks explain, to some extent, the persistence of a notary management model, with little adherence to the concrete needs of patients [55].

Only the evaluation and identification of priorities, which enables the construction of a health care model focused on primary health care that can also cope with chronic diseases and conditions, will allow innovation in care. This, in turn, will allow the implementation of new care strategies, whether individual or collective, and the transformation of health care in this region.

## Abbreviations

ACIC: Assessment of Chronic Illness Care
CCM: chronic care model
IPAQ: International Physical Activity Questionnaire
MET: metabolic equivalent of task
ODK: open data kit
SAPPA: Study of Health in Primary Care for the Amazonas Population
STROBE: strengthening the reporting of observational studies in epidemiology
T2DM: type 2 diabetes mellitus

## Data Availability

The data that support the findings of this study will be available on request from the corresponding author, EBL. The data will not be publicly available due to personal identification restrictions that could compromise the privacy of research participants.
